# Blood Vessel-Derived Acellular Matrix for Vascular Graft Application

**DOI:** 10.1155/2014/685426

**Published:** 2014-07-16

**Authors:** Luigi Dall'Olmo, Ilenia Zanusso, Rosa Di Liddo, Tatiana Chioato, Thomas Bertalot, Enrica Guidi, Maria Teresa Conconi

**Affiliations:** ^1^Istituto Oncologico Veneto, 35131 Padua, Italy; ^2^Department of Pharmaceutical and Pharmacological Sciences, University of Padua, Via Marzolo 5, 35131 Padua, Italy

## Abstract

To overcome the issues connected to the use of autologous vascular grafts and artificial materials for reconstruction of small diameter (<6 mm) blood vessels, this study aimed to develop acellular matrix- (AM-) based vascular grafts. Rat iliac arteries were decellularized by a detergent-enzymatic treatment, whereas endothelial cells (ECs) were obtained through enzymatic digestion of rat skin followed by immunomagnetic separation of CD31-positive cells. Sixteen female Lewis rats (8 weeks old) received only AM or previously *in vitro* reendothelialized AM as abdominal aorta interposition grafts (about 1 cm). The detergent-enzymatic treatment completely removed the cellular part of vessels and both MHC class I and class II antigens. One month after surgery, the luminal surface of implanted AMs was partially covered by ECs and several platelets adhered in the areas lacking cell coverage. Intimal hyperplasia, already detected after 1 month, increased at 3 months. On the contrary, all grafts composed by AM and ECs were completely covered at 1 month and their structure was similar to that of native vessels at 3 months. Taken together, our findings show that prostheses composed of AM preseeded with ECs could be a promising approach for the replacement of blood vessels.

## 1. Introduction

Cardiovascular diseases (CVDs) represent the leading cause of death in the western countries [1]. Since pharmacological treatment mainly consisting in antiplatelet drugs and cholesterol-lowering agents (statins) has been proven to be often not sufficient [2], the implantation of a vascular graft may be needed. Although autologous vessels, such as the internal mammary artery, radial artery, or the saphenous vein, are considered the golden standard for replacement of malfunctioning or diseased blood vessels, their availability is limited especially in elderly patients [3]. Synthetic materials, such as Dacron and expanded polytetrafluoroethylene (ePTFE), perform reasonably in high-flow, low-resistance conditions and they can be used successfully to substitute large diameter vessels [4]. Nevertheless, they are not as suitable for small diameter (<6 mm) arterial grafts due to their thrombogenicity [5]. Thus, the development of an effective vascular graft, possessing biomechanical properties matching those of native vessel, has become one of the main targets of tissue engineering.

Three approaches have been designed for vascular regeneration: (i) biodegradable synthetic polymer-based constructs, (ii) cell self-assembly blood vessels, and (iii) decellularized tissue grafts.

Several polymers have been used to obtain tissue-engineered blood vessels (TEBVs): poly(dimethylsiloxane), poly(caprolactone), poly(methyl methacrylate), poly-L-lactic acid (PLLA), polyglycolic acid, poly(glycerol sebacate), and polyvinyl alcohol (PVA) [6, 7]. Notably, Shin'oka et al. [8] carried out the first human clinical study enrolling 42 patients who underwent cavopulmonary connection with a TEBV composed of a copolymer of *ε*-polycaprolactone-polylactic acid reinforced with woven polyglycolic acid previously seeded with autologous bone marrow-derived mononuclear cells. Although some grafts presented stenosis, long-term results at 2 years indicated that TEBV functioned well without any aneurysm or graft rupture.

Another approach for the fabrication of TEBVs is cell self-assembly developed by L'Heureux and colleagues [9] who used entirely autologous cells cultured* in vitro*. Sheets of smooth muscular cells (SMCs) and fibroblasts were grown to overconfluence and then assembled over a mandrel to form a tubular structure that was cultured for 6–8 weeks. During this incubation period, the autologous cells arranged themselves circumferentially producing large amounts of extracellular matrix (ECM). Recently, promising results were obtained after implantation of these TEBVs into 10 patients receiving hemodialysis with failed arteriovenous fistulas [10, 11]. Although self-assembly grafts possess excellent mechanical properties and nonimmunogenic nature, a very long culture period (about 8–10 weeks) is required to obtain an implantable construct.

Finally, decellularized tissues, named also acellular matrices (AMs), can be obtained from various anatomical sites through several procedures involving both physical and chemical agents, such as surfactants and enzymes [12]. These biomaterials possess a preformed structurally organized ECM containing angiogenic growth factors, such as b-FGF and VEGF [13, 14], and lacking immunogenic issues. Small intestinal submucosa (SIS), porcine carotid artery, aorta, and even canine carotid arteries have been evaluated using animal models [15, 16]. Beside the potential risk of viral transmission from animal tissue, the implantation of decellularized xenografts gave disappointing results. Indeed, the treatment of patients with decellularized bovine ureters resulted in high failure rate probably due to the presence of residual immunogenic contaminants, such as galactose-alpha-1,3-galactose (alpha gal) [17]. Thus, the use of homologous AMs seems to be a more suitable approach for vascular replacement.

Overall, the major drawbacks connected to described TEBVs are thrombogenicity, the occurrence of intimal hyperplasia, the progressive atherosclerotic degeneration, and the time required for culturing the cells. Starting from these considerations, the present study developed AM-based vascular grafts composed of AMs derived from iliac arteries and skin microvascular ECs and evaluated their effectiveness as abdominal aorta interposition grafts in Lewis rats.

## 2. Materials and Methods

### 2.1. Materials

Phosphate-buffered saline (PBS) tablets were purchased from Gibco Invitrogen Corp. (Paisley, UK). Rabbit monoclonal anti-MHC I and II and rabbit polyclonal anti-von Willebrand factor primary antibodies were provided by Abcam (Cambridge, UK). Horse pan-specific secondary antibody, DAB Peroxidase Substrate, Fluorescein Avidin DCS, and Vectashield Mounting Medium were from Vector Laboratories (Burlingame, CA, USA). Collagenase B and Dispase II were obtained from Roche Applied Science (Indianapolis, IN, USA). The Endothelial Cell Growth Medium MV2 was purchased from PromoCell GmbH (Heidelberg, Germany). Cell strainer, tissue culture-treated dishes, and fibronectin were from BD Biosciences (San Jose, CA, USA). Mouse monoclonal anti-rat-CD31 antibody was provided by Millipore (Billerica, MA, USA). Dynabeads M-450 were obtained from Life Technologies (Monza, Italia). Movat pentachromic stain kit was from Diapath S.p.A. (Martinengo, Italy). Contramal was purchased by Grünenthal (Aachen, Germany), whereas Terramicina was from Phibro Animal Health Corporation (Teaneck, NJ, USA). All other chemicals and reagents were provided by Sigma-Aldrich (St. Louis, MO, USA).

### 2.2. Animals

All procedures described and animal protocols were approved by the Institutional Animal Care Committee of the University of Padua and by the Italian Health Department. Lewis rats (8 weeks old, 200–300 g body weight) were purchased from Charles-River (Como, Italy). Male animals were sacrificed using CO_2_ inhalation and iliac arteries as well as dermis were collected and rinsed with PBS.* In vivo* experiments were carried out on female rats.

### 2.3. Acellular Matrices

AMs were prepared by Meezan et al. method [18] with minor modifications. Briefly, iliac arteries were processed with distilled water for 72 h at 4°C, 4% sodium deoxycholate for 4 h, and 2,000 kU deoxyribonuclease I (DNase-I) in 1 M NaCl for 3 h. The treatment was repeated twice till the cells were completely removed. The endothelium was detached incubating the vessels with collagenase IV (0.05% in PBS) at 37°C for 1 min. To verify the lack of cells, AMs were fixed with 10% formalin in PBS, paraffin-embedded, and stained with hematoxylin/eosin (H/E). On the other hand, the absence of cellular membrane residuals was evaluated by immunoistochemistry. Briefly, slices were treated with hydrogen peroxide for 30 min at room temperature (RT) and nonspecific binding sites were blocked with 10% bovine serum albumin (BSA) in PBS for 20 min at RT. Samples were incubated for 60 min with rabbit monoclonal anti-MHC I and II primary antibodies (1 : 500 in 4% BSA in PBS) and then with the horse pan-specific secondary antibody (1 : 8 in 4% BSA in PBS) for 30 min at RT. The reaction was developed with DAB Peroxidase Substrate following the manufacturer's instruction. Finally, nuclei were counterstained with hematoxylin. Negative controls were obtained by omitting the primary antibody. Alternatively, AMs were fixed with 4% glutaraldehyde in 0.1 M cacodylate buffer (pH 7.2) for 24 h and dehydrated with 70% and 90% ethanol (2 h each) and 100% ethanol overnight. After critical point drying and gold sputtering, samples were examined by a scanning electron microscope (SEM; Stereoscan-205 S, Cambridge, UK) using a standard protocol.

### 2.4. Cell Cultures

Dermis, obtained from the abdomen of male rats, was rinsed in PBS, minced, and treated with 0.25% Collagenase B and 0.25% Dispase II for 1 h at 37°C. The digested tissue was filtered through a 100 *μ*m cell strainer. Cell suspension was centrifuged and resuspended in culture medium MV2. Cells were then seeded on fibronectin- (1 *μ*g/cm^2^) coated dishes and cultured at 37°C with 5% CO_2_. To obtain pure skin microvascular ECs, cultures grown to 80% confluence were immunoseparated using Dynabeads M-450 previously coated with the mouse monoclonal anti-rat-CD31 antibody following manufacturer's instruction [19]. Briefly, cells were incubated with magnetic beads for 30 min at 4°C (5 beads/cell). ECs bound to the coated beads were collected with a magnetic particle concentrator and unbounded cells were removed by means of 2 washes with culture medium. Finally, ECs were seeded on fibronectin-coated dishes, cultured with MV2 medium, and used until the 4th passage. The isolated cells were characterized by immunofluorescence performing with rabbit polyclonal anti-von Willebrand factor (1 : 400). Briefly, cells were fixed with 4% formalin for 10 min at 4°C, washed in PBS, and incubated at RT for 1 h with 10% horse serum in PBS and, for 1 h, with the primary antibody. After rinsing with PBS, cells were treated with the horse pan-specific secondary antibody (1 : 8) for 30 min and with Fluorescein Avidin DCS 1 : 500 in HEPES 10 mM and NaCl 0.15 M for 10 min. Samples were mounted with mounting medium with DAPI.

### 2.5. Cultures of ECs on AMs

Under static conditions, ECs (4 × 10^5^/cm^2^) were seeded onto the luminal surface of AMs, previously incubated with MV2 medium for 3 h at 37°C. Cultures were maintained for 72 h in MV2 medium and then fixed for morphological analysis or* in vivo* implanted.

### 2.6. *In Vivo* Experiments

Female Lewis rats (8 weeks old) were divided into two groups according to the kind of the implanted graft: group 1 AMs (*n* = 7) and group 2 AMs plus ECs (*n* = 9). Under isoflurane anesthesia (3% isoflurane carried by oxygen, 1 L/min), the abdominal area was shaved and aseptically prepared using povidone-iodine (Betadine). The muscles were exposed with a 3 cm abdominal incision and, after peritoneal incision, animals received analgesic (5 mg/Kg Tramadol, Contramal) intraperitoneally. The abdominal aorta was exposed and isolated and, after clamping the vessel, a segment of aorta was excised and the graft (about 1 cm in length) was anastomosed proximally and distally end-to-end using continuous 10.0 polypropylene sutures. Animals received antibiotic (Terramicina, 60 mg/kg) on the 3rd and 6th days after surgery and Contramal for 3 days postoperatively. No anticoagulants or antiplatelets were administered postoperatively. Animals were sacrificed by CO_2_ inhalation either 1 (group 1 *n* = 4; group 2 *n* = 4) or 3 months (group 1 *n* = 3; group 2 *n* = 5) after implantation. The implants were recovered and each sample was divided into two pieces: one was fixed with 4% glutaraldehyde in 0.1 M cacodylate buffer for SEM and processed as described above and one was fixed in 10% neutral buffered formalin and paraffin-embedded. Five *μ*m thick sections were treated with Movat pentachromic stain kit according to the manufacturer's instruction. This stain produces purple-black elastic fibers and nuclei, blue to green mucins, red muscle, and red fibrinoid against a yellow background with collagen.

## 3. Results and Discussion

**Figure 1:**
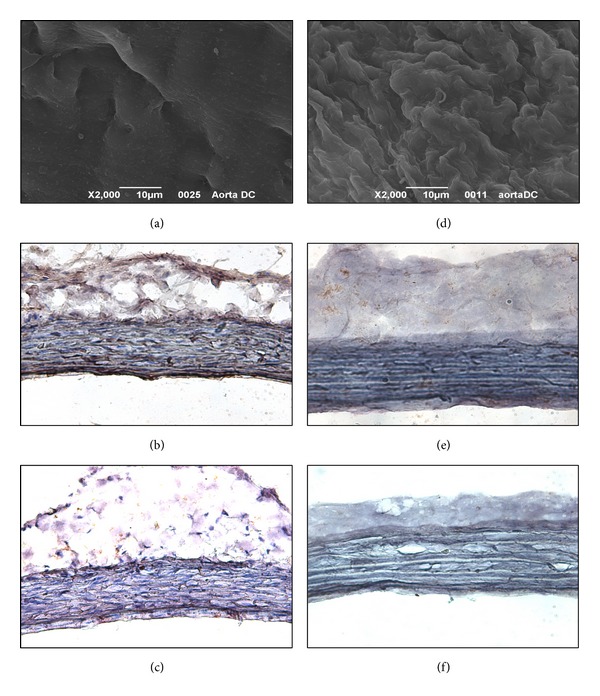
Iliac arteries before (a–c) and after (d–f) the decellularization treatment. (a), (d) SEM micrographs of the luminal sides. Immunoreactivity against MHC I (b, e) and II (c, f) antigens stains brown (magnification ×200).

The ideal biomaterial for the generation of TEBVs should not only possess the mechanical properties of the native vessels but also promote* in vivo* a regenerative response through the induction of host cell ingrowth. In this context, AMs, obtained by a detergent-enzymatic treatment, have been proven to support* in vitro* adhesion, growth, and function of several cell types [20–22]. Furthermore, the decellularization process induces the loss of the major histocompatibility complex markers but maintains angiogenic factors, such as b-FGF and TGF-*β* [13, 23, 24]. Thus, AMs can present angiogenic activity, an important factor for the* in vivo* integration of the tissue substitutes. Indeed,* in vivo* AMs act as a template allowing the host cell ingrowth and they are remodeled in a living tissue [25, 25–27]. Moreover, they represent preformed structures whose length and gauges can be chosen according to the dimension of the segment to be repaired. Another advantage is the possibility to have easy and unlimited availability of inexpensive grafts containing tissue-specific proteins. Herein, two cycles of detergent-enzymatic treatment were needed to completely remove cells from iliac arteries (Figure 1). To detach the endothelial layer (Figure 1(a)), still present at the end of the first cycle, a treatment with collagenase IV was needed between the two decellularization cycles (Figure 1(d)). The structure of the native vessels was well preserved in AMs that lacked both MHC I (Figure 1(e)) and II (Figure 1(f)) cell membrane antigens, normally present in native tissue (Figures 1(b) and 1(c)).

To guarantee an* in vivo* long-term patency of TEBVs, intimal hyperplasia and graft occlusion must be avoided. To achieve this goal, a continuous lining of ECs on the luminal surface of TEBVs seems to be essential since it represents a physical barrier that is able to prevent platelet adhesion and the activation of the coagulation cascade [28]. Furthermore, cell seeding reduces the overall influx of macrophages and the magnitude of M1 activation, avoiding scar formation [29]. These effects, in turn, lead to a functional remodeling of the vessel wall. In particular, elastic fibers play a pivotal role as they determine the mechanical properties of both high and small resistance vessels, thus preventing stenosis [30].

**Figure 2:**
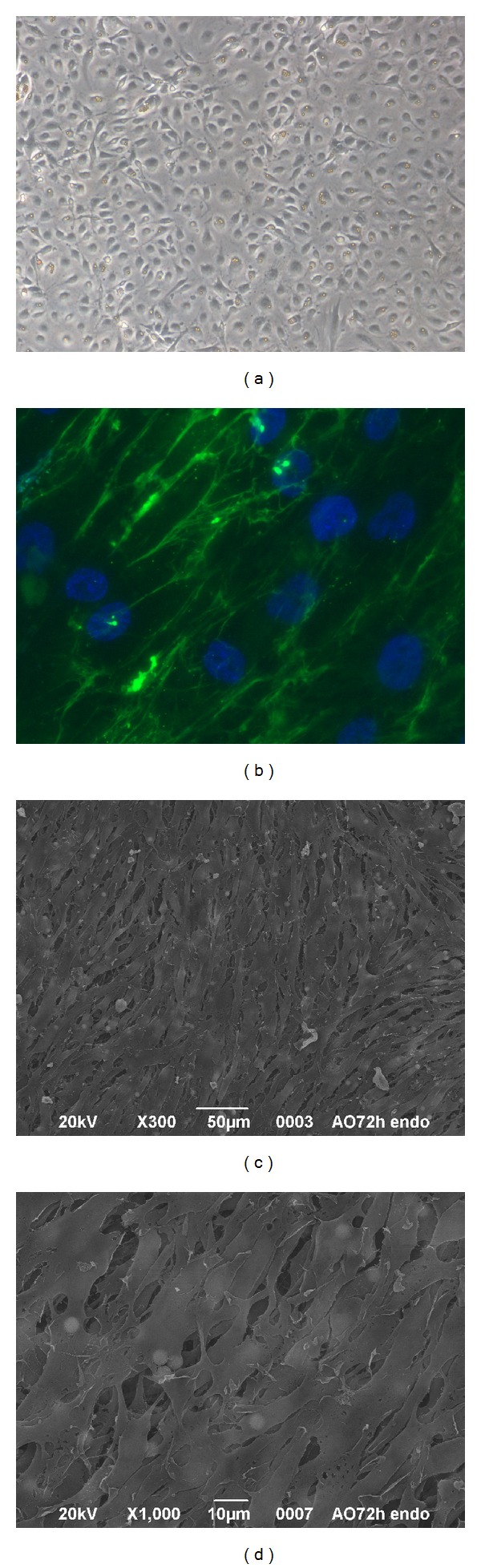
Cultures of skin microvascular ECs on tissue culture treated plates (a, b) and AMs (c, d). (a) Phase-contrast microscopy (magnification ×100). (b) Immunofluorescence carried out using anti-von Willebrand factor antibody (magnification ×400). (c), (d) SEM micrographs of ECs/AM cultures at 72 h from seeding.

Autologous ECs harvested from blood vessel, such as veins, are terminally differentiated, have limited proliferation potential, and lose their function during* in vitro* expansion [31]. Alternatively, bone marrow-derived mononuclear mesenchymal stromal cells (BM-MSCs) [32] and endothelial progenitor cells (EPCs) [33, 34] have been used to make grafts hemocompatible. Nevertheless, the use of these cells can be limited by their low number in the adult tissues, low proliferative rate, and the invasive procedures needed to obtain them, leading to morbidity for the donors. Furthermore, BM-MSCs may induce calcification and thrombus formation [35]. In this work, microvascular ECs were obtained from skin biopsy; that is, a moderately invasive procedure that avoids the need to remove healthy vessels. ECs presented a polygonal shape (Figure 2(a)) and maintained the endothelial phenotype till the fourth culture passage as demonstrated by the expression of von Willebrand factor (Figure 2(b)). Furthermore, at 72 h from seeding onto AMs, they formed an almost continuous monolayer on the luminal surface of AMs (Figures 2(c) and 2(d)) and maintained their immunoreactivity to anti-CD31 antibody (data not shown). Notably, starting from a 1 cm^2^ skin fragment, 6 × 10^6^ ECs were obtained in about 10 days, allowing the production of an implantable graft within less than two weeks. This period may represent a clinically relevant time frame. Other vascular regeneration techniques already used in clinical practice take about 4–6 weeks to generate TEBVs [4].

**Figure 3:**
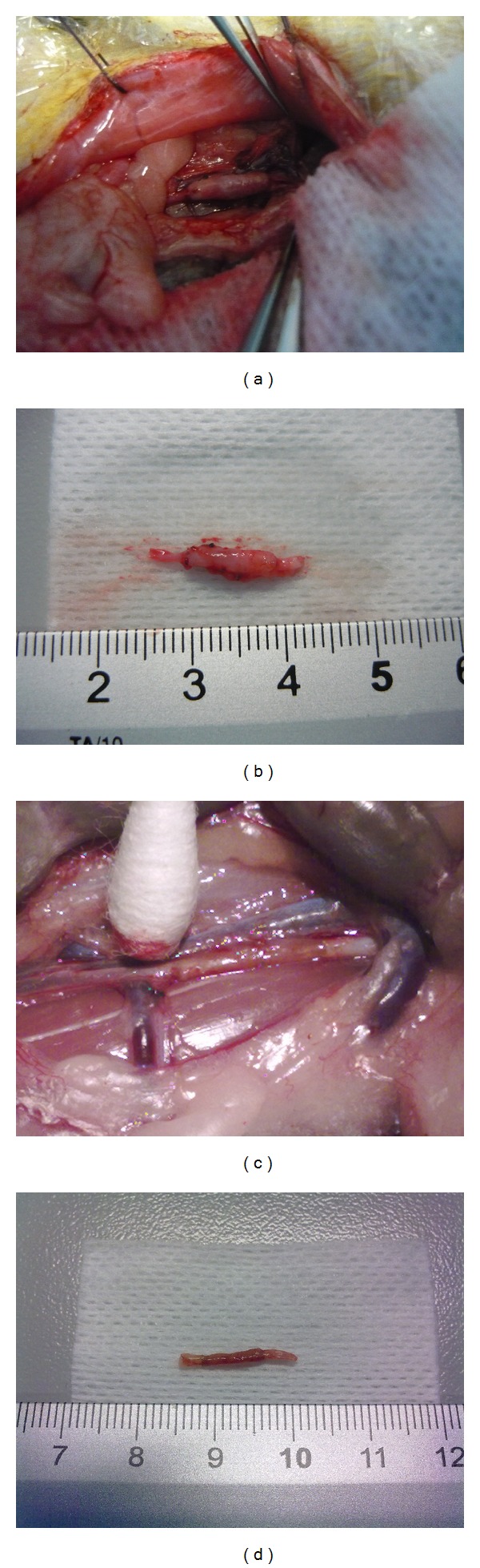
Implants composed of AMs (a, b) and ECs and AMs (c, d) 3 months after surgery.

**Figure 4:**
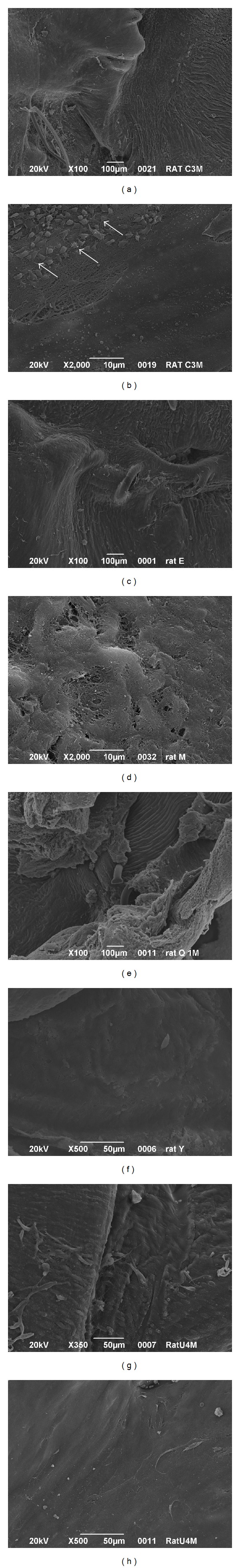
SEM micrographs of the luminal sides of AMs (a–d) and ECs/AM grafts (e–h) at 1 (a, b, e, f) and 3 (c, d, g, h) months after surgery. Arrows indicate area covered by platelets.

**Figure 5:**
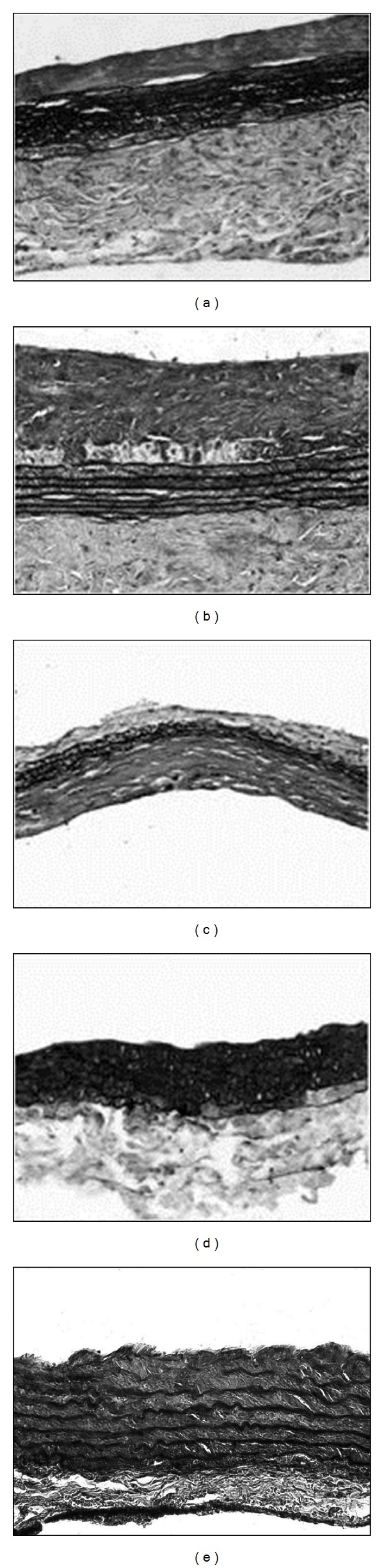
Movat staining of AMs (a, b) and ECs/AM (c, d) grafts after 1 (a–c) and 3 (b–d) months from surgery (magnification ×200). (e) Native aorta.

As expected, the implantation of only AMs into the abdominal aorta of female Lewis rats gave unsatisfactory results. Although all animals survived, at both 1 and 3 months (Figures 3(a) and 3(b)), explanted grafts presented higher external diameters than those of host vessels. One month after surgery, only the borders of the patch were reendothelialized (Figure 4(a)), whereas, in the other areas, several platelets adhered to the exposed collagen fibers (Figure 4(b)). Only one animal did not present thrombi inside the implanted AM. At 3 months, the luminal surface was almost completely covered by ECs (Figures 4(c) and 4(d)) and no thrombi were detected. At both time points, although elastic fibers were well organized, neointimal hyperplasia and thickening of the adventitial layer were evident (Figures 5(a) and 5(b)). Furthermore, the adventitia of implanted AM grafts presented a more fibrous structure than that observed in the native aorta (Figure 5(e)). The latter evidence agrees with the findings of Assmann et al. [36], who transplanted homologous decellularized aortic conduits in the infrarenal aorta of rats. Although the grafts remained patent for 8 weeks, hyperplastic tissue formation and implant microcalcification occurred. Neointimal hyperplasia was also detected in decellularized and heparinized grafts implanted in dogs as carotid artery bypass grafts [16].

The* in vitro* coverage of the luminal side of AMs with skin microvascular ECs greatly improved the outcomes of the reconstructive surgery avoiding thrombus formation and allowing good patency. It has been already demonstrated that autologous cells, rather than to mask platelets adhesion sites, may produce soluble factors that are able to enroll the cells from the neighbouring host tissues [37]. Furthermore, the seeded cells may lead to the homing of circulating monocytes [29] that, in turn, release the monocyte chemotactic protein-1, stimulating the regeneration of the blood vessel [38]. Although it has not been verified whether the seeded ECs were present on the implanted grafts, we can suppose that the initial endothelial coverage could be transient and progressively replaced by the host cells [39]. At both time points, the external diameter of the explanted grafts was similar to the one of host aorta (Figures 3(c) and 3(d)). One month from surgery, all luminal surfaces were completely reendothelialized and no signs of platelet adhesion were visible (Figures 4(e)–4(h)). The implanted grafts appeared to be remodelled during the time (Figures 5(c) and 5(d)). Indeed, at 1 month, histological analysis revealed a moderate hyperplasia of the tunica intima that disappeared at 3 months. Furthermore, the thickness of the elastic layer increased over the time. Similar results were obtained by Leyh et al. [40] who implanted TEBVs composed of homologous decellularized arteries with or without autologous ECs in the pulmonary circulation of sheep. The* in vitro* reendothelialized conduits performed well for as long as 6 months, whereas the lack of ECs led to aneurysm formation.

## 4. Conclusions

Herein, we show that AM-based TEBVs lead to unsatisfactory results since the lack of ECs contributes to graft thrombogenicity and promotes intimal proliferation. One month after surgery, the luminal surface of implanted AMs was partially covered by ECs and several platelets adhered in the areas lacking cell coverage. Intimal hyperplasia, already detected after 1 month, increased at 3 months. On the contrary, the* in vitro* reendothelialized AMs led to well-performing vascular conduits whose structure resembles that of the host vessel. The ECs lining the luminal surface of the grafts function as a barrier preventing the platelet adhesion on extracellular matrix, and they may influence, in a paracrine manner, the regeneration process by recruiting the host cells and modulating the inflammatory response. In agreement with this statement, our results demonstrated that only the implanted ECs seeded grafts were progressively changed with a thickening of the tunica media. Thus, the cellular component of the graft allows AMs to act as a temporary template that can be correctly remodelled by the host cells in a functional tissue.
